# Supplementary material for: Are carbon emissions trading and green financial instruments synergistic? -Comprehensive quantitative research based on content analysis
Source: PLoS One. 2024 Mar 7;19(3):e0298601. doi: 10.1371/journal.pone.0298601 (PMC10919601; doi:10.1371/journal.pone.0298601)
Supplement: S1 Data — (ZIP) [file pone.0298601.s001.zip › Data/地级市Z_F工作报告.zip!/╜¡╦╒/╦╒╓▌╩╨/└┤╫╘╣1⁄2╓┌║┼í╛┬φ┐╦╩2╛▌═°í┐_2013.docx]

各位代表：

　　现在，我代表苏州市人民政府，向大会报告工作，请予审议，并请市政协委员和其他列席人员提出意见。

　　一、2012年工作回顾

　　过去的一年，市政府在中共苏州市委的正确领导下，团结依靠全市人民，紧扣科学发展主题和转变经济发展方式主线，围绕加快“两个率先”、建设“三区三城”的奋斗目标，积极应对国内外环境的复杂变化，深入实施创新引领、开放提升、城乡一体、人才强市、民生优先、可持续发展战略，突出加快转型、稳定外贸、兴盛文化、广惠民生等重点，统筹做好各项工作，推动了经济平稳较快发展和社会全面进步。

　　1．经济保持平稳增长，转型升级成效明显。全市完成地区生产总值1.2万亿元，比上年增长10%；地方公共财政预算收入1204亿元，增长9.4%；全社会固定资产投资5265亿元，增长17%。工业经济提档升级，实现总产值3.44万亿元，增长3.3%；其中规模以上工业产值2.87万亿元，增长5%。制造业领域新兴产业实现产值1.23万亿元，增长11%，占规模以上工业的比重达到43%，比上年提高5个百分点。提升发展支柱产业，实施万企升级计划，技术改造投资占工业投资的比重达到68.7%。吴江经济技术开发区成为国家新型工业化产业示范基地。服务业量质齐升，增加值占地区生产总值的比重比上年提高1.5个百分点。新增市级服务业集聚区10个、制造业分离发展服务业企业260家。金融业规模持续扩大，金融总资产达到2.7万亿元，增长18%。商贸业稳步增长，完成社会消费品零售总额3254亿元，增长15%。旅游业快速发展，接待境内外游客人次、旅游总收入分别增长10%和17%。金鸡湖景区成功创建国家5A级景区。文化产业创新发展，张家港软件（动漫）产业园成为国家影视网络动漫实验园，相城区阳澄湖数字文化创意产业园二期建成启用，首届文化创意设计产业交易博览会成功举办。大力推进农业现代化，太仓市、相城区进入国家现代农业示范区行列，全市现代农业园区面积增加到47.3千公顷。粮食单产、总产、效益全面增长，省下达的扩储计划顺利完成。

　　积极构建区域创新体系，科技综合实力保持全省领先。加大创新投入力度，全社会研究与试验发展经费支出占地区生产总值的比重提高到2.6%。实施高新技术产业倍增工程，新增高新技术企业588家，实现产值1.17万亿元，增长11%。大中型工业企业研发机构建有率提高到80%，新增4个国家级企业技术中心。苏州纳米城首期建成使用，苏州生物医学工程技术研究所纳入中科院序列，长三角地区首个国家科技成果转化服务示范基地落户我市。国家“千人计划”创业投资中心暨沙湖股权投资中心二期加快建设，“科贷通”项目新增贷款34.8亿元。国家专利审查协作江苏中心投入运行，我市成为首批国家知识产权示范城市，专利申请量、授权量均居全国大中城市第一位，又添中国驰名商标17件。我市被列为全国首批创建质量强市示范城市和综合服务标准化试点城市。实施各项人才计划，扩大国际精英创业周、“赢在苏州”创新创业大赛的品牌效应，新增国家“千人计划”人才51人，累计达到105人，继续位居全国地级市首位；新增省“双创计划”人才96人，累计达到301人，保持全省第一。

　　2．改革开放不断深化，发展活力持续增强。实施行政区划重大调整，撤销平江、沧浪、金阊区，合并设立姑苏区，成立苏州国家历史文化名城保护区；撤销吴江市，设立吴江区。省直管县体制改革试点和经济发达镇行政管理体制改革试点稳步开展。事业单位分类改革扎实推进。政府办基层医疗卫生机构全部配备、使用并零差率销售基本药物。加强监督管理，优化资源配置，国有资产运行质量和效益不断提高。开展营业税改征增值税试点，结构性减税步伐加快。注重金融创新，新增金融机构75家，总数达到605家，东吴人寿保险股份有限公司开业运营；新增上市企业12家，累计达到82家；成功发行全国首单中小企业私募债和科技型中小企业集合票据。民营经济进一步壮大，私营企业和个体工商户注册资本总额突破8700亿元。

　　全力以赴稳定外贸增长，优化开放型经济发展格局。全市完成进出口总额3060亿美元，增长1.6%以上，其中出口1748亿美元，增长4.4%以上。服务外包接包合同额、离岸执行额分别增长56.8%和52%。全国首个检验监管综合改革试验区获批设立，首个加工贸易废料交易平台建成运行。招商引资量稳质升，实际利用外资90.8亿美元，服务业、新兴产业利用外资分别占到33%和38.5%。中方境外协议投资额达到12亿美元，增长71.3%，总量实现全省“九连冠”。吴中经济开发区升格为国家级开发区，张家港保税港区汽车整车进口口岸获得国务院批准，苏州高新区成为全国首个国家知识产权服务业集聚发展试验区，苏州工业园区被列为国家进口贸易促进创新示范区，中德（太仓）中小企业合作示范区获批设立，太仓港成为全国第一个享受海港待遇的内河港口，苏州工业园区—相城区合作经济开发区建设大力推进。南北挂钩合作与对口援建工作积极开展。外事、侨务、对台工作再创新业绩，第二届中非民间论坛在我市成功举办。

　　3．城乡建设统筹推进，区域发展更趋协调。苏州市城市总体规划、轨道交通线网规划、太湖国家旅游度假区总体规划完成调整修编工作，东部综合商务城、西部生态科技城、南部太湖新城、北部高铁新城高标准建设。苏州火车站南站房和南广场配套工程基本完工。轨道交通1号线建成运营，2号线实现“轨通”，昆山花桥与上海的轨道交通对接进入铺轨阶段。苏虞张快速通道北段建成通车。苏州港新增9个万吨级以上泊位，完成货物吞吐量4.3亿吨、集装箱吞吐量587万标箱，分别增长12.4%和25.2%，其中太仓港集装箱吞吐量突破400万标箱。张家港复线船闸实现通航。±800千伏锦苏特高压输电线路及同里换流站建成投运。新增人防设施72万平方米。垃圾焚烧发电三期工程顺利竣工，餐厨垃圾收集处置二期和七子山垃圾填埋场二期工程基本建成。强化市容环境、道路交通管理，打造最干净、最整洁城市取得成效。

　　城乡一体化发展综合配套改革全面深化，农村发展更具活力。城乡空间布局不断优化，48%的农民实现集中居住，90%的工业企业进入工业园，88%的承包耕地实现规模经营。农村集体经济继续壮大，集体资产突破1200亿元，村均收入达到580万元，分别增长14.2%和15%。新增新型合作经济组织431个，持股农户比例提高到96%。城乡养老保险、居民医疗保险全面并轨。公共财政投入“三农”的总量、增量和比例持续提高，政策性农业保险和农业担保覆盖面逐步扩大。做好全国水利现代化试点工作，加强农村水利和圩区堤防达标建设，防洪抗灾能力进一步增强。在全省率先完成村庄环境整治任务，长效管理机制初步建立。

　　4．节能减排力度加大，生态环境不断优化。加强水源地保护，集中式饮用水源地水质达标率保持100%。太湖流域水污染治理年度任务全面完成，东太湖综合整治和走马塘拓浚延伸工程基本完工。实施水质提升工程，干河清淤城区河道110条，整治农村黑臭河道244条。新建和改造污水处理厂11座，新增污水处理能力20.8万吨，建设配套截污管网799公里，城镇生活污水处理率达到95%，农村生活污水处理率达到60%，其中太湖、阳澄湖保护区分别达到80%和78%。实施蓝天工程，开展PM2.5监测，空气质量有所改善。防控噪声污染，声环境功能区噪声达标率保持100%。加强核与辐射安全监管，规范固体废物处置，切实防治重金属污染。狠抓节能减排，淘汰落后产能，单位地区生产总值能源消耗下降、主要污染物排放量削减完成省下达任务。落实最严格的耕地保护制度和占补平衡制度，土地利用水平明显提高。实施循环经济发展规划，再生资源综合利用体系和循环产业链加快构建。加强节能监察审计与合同能源管理，开展“能效之星”创建活动，重点企业能源消耗继续下降。建成石湖滨湖景区等重大绿化工程，市区新增绿地450万平方米。全市新增林地绿地13.3千公顷，陆地森林覆盖率达到27.8%。高度重视湿地保护和管理，恢复湿地650万平方米。我市获批开展国家低碳城市试点工作，苏州及各县级市、区全部成为全国生态文明建设试点地区，国家环保模范城市复核实现“满堂红”。

　　5．惠民措施有效落实，民生水平得到提高。城镇居民人均可支配收入、农民人均纯收入分别达到3.8万元和2万元，增幅均在14%以上。年度实事项目全面完成。新增就业16.8万人，免费培训城乡劳动者43.8万人，城镇登记失业率控制在2.7%，苏州籍高校毕业生就业率达到96.6%，零就业和零转移贫困家庭保持动态清零，我市被评为全国创业先进城市。城镇职工养老、医疗、失业、工伤、生育五大社会保险参保覆盖面和基金征缴率均保持在99%以上，吴中、相城区社会保险与市本级实现并轨，太仓市大病再保险、吴江区特殊病症人员再救助等工作取得实效。城乡居民最低生活保障指导标准由每人每月500元提高至570元。苏州社会保障·市民卡顺利发行。全市发放各类社会救助资金18.3亿元，惠及困难群众16.8万人次。市社会福利总院一期工程如期竣工。整体推进养老服务，新增养老床位8355张、日间照料中心87个、助餐点142个。发展残疾人服务事业，我市进入全国首批“阳光家园”示范区行列，贫困残疾人家庭普及基本家用电器计划积极实施。开展接送流浪孩子回家专项行动，基本实现城市街面无流浪未成年人目标。红十字和公益慈善事业健康发展。新开工保障性住房3.7万套，竣工2.5万套，为4894户困难家庭发放廉租住房租赁补贴。市区完成113万平方米老住宅小区和16万平方米零星居民楼整治任务，实施城区居民家庭“改厕”8739户，解危修缮直管公房4.5万平方米，启动改造城中村（无地队）17个。南环新村解危改造新建住宅封顶。全市新增参加住房公积金制度的职工52.6万人，职工使用住房公积金187.6亿元。落实公交优先发展新三年行动计划，市区新购公交车506辆，新增电调专用出租车300辆，增加公共自行车网点151个。开展食品安全集中整治“天安行动”和药品安全示范市创建活动，建成食品安全监测预警体系。新建农产品平价直销点102个、“菜篮子”直供店46个。居民消费价格总水平涨幅控制在2.7%。

　　6．各项事业繁荣进步，社会管理全面加强。新建和改扩建中小学、幼儿园137所，新增公办学位7.4万个，基本完成中小学校舍安全工程。我市普通高中高考录取率位居全省第一。市高等职业技术学校、旅游与财经高等职业技术学校改扩建工程全面完成，教育惠民服务中心、学生资助管理中心、教师发展中心相继成立。与中外著名高校合作办学取得新成果。完善公共文化基础设施和服务网络，国家公共文化服务体系示范区创建工作积极推进，张家港市在全国率先推出网格化公共文化服务模式。一批历史遗存被列入中国大运河申报世界文化遗产名单，昆曲遗产保护、继承、弘扬工程通过国家文化创新工程验收。滑稽戏《青春跑道》获得国家文化部优秀保留剧目大奖。虎丘地区综合改造、桃花坞历史文化片区综合整治保护利用工程加快推进，相门、平门、阊门段古城墙保护性修复顺利完成，古建老宅、古村落保护修缮取得成效。《苏州市志》完成总纂，《苏州通史》抓紧编写。健全城乡社区卫生服务体系，镇村卫生机构全部达到省定建设标准。医院与基层医疗卫生机构之间的双向转诊广泛实施，建成首个社区影像远程会诊中心。市区集约式电话预约挂号“一号通”平台投入使用。常熟市、吴江区成为国家卫生应急综合示范市（区）。城乡一体的人口和计划生育管理体系不断完善，生育关怀、科学育儿、流动人口服务管理等工作有序开展。苏州体育健儿在伦敦奥运会、残奥会上争金夺银、再创佳绩，我市获得2015年世乒赛举办权。全民健身服务积极开展。民族团结进步事业健康发展，依法管理宗教事务水平不断提高。妇女儿童事业继续走在全国前列。支持驻苏部队建设，做好优抚安置工作，军政军民关系更加密切。

　　精神文明建设深入推进，各项社会管理成效显著。苏州和张家港市在全国城市文明程度指数测评中获得可喜成绩，均被评为全国未成年人思想道德建设工作先进城市。加强法治苏州建设，昆山市成为全省首批法治建设示范市，7个县级市、区被评为省法治县（市、区）创建工作先进单位。法律援助、“关爱民生法治行”等工作有效开展。吴江区成为全国普法和依法治理联系点。太仓市“政社互动”经验得到推广。张家港、太仓、昆山市和吴江区获评全国村务公开民主管理示范单位。实施“万企贯标、百企示范”和“诚信计量”工程，社会信用体系加快构建。强化网格化社会管理综合治理，安定和谐的社会秩序得到维护。实行流动人口居住证制度，与人口主要流出地建立全面协作机制。做好大排查大调解工作，层层落实信访工作责任，调处解决了一批矛盾纠纷和信访问题。切实增强应急管理能力，突发公共事件得到妥善处置。狠抓安全生产专项整治、重大隐患挂牌整改和“打非治违”工作，有效遏制了重特大事故。

　　与此同时，政府自身建设进一步加强。认真做好政府立法工作，提请市人大常委会审议通过地方性法规2件，颁布政府规章3件，制定规范性文件5件。扎实开展助企解忧、惠民服务、项目绩效、服务提质、品牌引领等作风效能建设“五大行动”，健全行政审批跟踪服务和行政权力公开透明运行机制，便民服务水平得到提升。完善惩治和预防腐败体系，严肃查处了一批违法违纪案件。严控行政经费，公车购置及运行、会议、公务接待、因公出国（境）支出普遍下降。开展金融行业专项监督检查，优化企业融资环境。加强审计监督，政府资金使用效率继续提高。市政府自觉接受市人民代表大会及其常务委员会的法律监督、工作监督和市政协的民主监督，坚持重大事项向市人大常委会报告、向市政协通报，注重发挥各民主党派、工商联、无党派人士和人民团体的参政议政作用。组织实施人大议案1件，办复人大代表建议307件、政协提案615件。

　　各位代表，过去的一年，面对宏观形势的严峻挑战，面对经济增长的下行压力，面对转型发展的迫切需要，全市人民齐心协力、和衷共济，付出艰辛努力，发挥聪明才智，克服种种困难，获得了来之不易的发展成果。在此，我代表市政府向全市人民，向人大代表、政协委员，向离退休老同志，向各民主党派、工商联和无党派人士，向各人民团体，向驻苏解放军、武警和消防官兵，向国家和省驻苏单位，向参与、支持、关心苏州现代化建设的海内外朋友，表示衷心的感谢和崇高的敬意！

　　回顾过去的一年，我们也看到，经济社会发展和政府工作中仍然存在不少问题与不足，主要是：受国际市场需求持续低迷等因素影响，开放型经济和实体经济面临更大困难，地区生产总值、地方公共财政预算收入、进出口总额、社会消费品零售总额增速趋缓；自主创新能力有待提高，产业结构亟需优化，节能减排任务艰巨，转变经济发展方式任重道远；环境质量不如人意，可持续发展能力还要切实增强；社会建设和社会管理需要进一步加强，政府工作效能和服务水平必须加快提升。对此，我们将高度重视，下大力气加以解决。

　　二、2013年工作总体要求和主要预期目标

　　当前，我们面临的外部环境依然错综复杂、充满变数。全球经济低速增长态势仍将延续，各种形式的贸易保护主义明显抬头，世界经济已由危机前的快速发展期进入深度转型调整期。国内经济趋稳的基础尚不稳固，扩大有效需求面临不少制约因素，企业生产经营困难增多，经济增长下行压力和产能相对过剩的矛盾有所加剧。在正视困难的同时，更应当看到的是，我国发展仍处于可以大有作为的重要战略机遇期。中共十八大明确了未来发展的新要求新任务，为我们做好今后工作指明了方向。国家加强和改善宏观调控，全面深化体制改革，积极稳妥推进城镇化，必将有力地促进经济持续健康发展。苏南现代化建设示范区的推进、苏州城市发展格局的优化也为经济增长提供了新的动力和支撑。综合分析，一方面，整体有利的宏观经济环境已经确立、总体向好的发展趋势已经确立、稳中求进的工作基调已经确立；另一方面，外需不足与内需不旺双重叠加、企业成本上升与产品价格下降双重挤压、财政收入增长趋缓与刚性支出持续上升双重影响等一系列矛盾仍然存在。我们将深刻认识苏州经济进入平稳增长期的显著特征，因势利导，主动作为，全力推动经济社会发展再创新辉煌。

　　根据中共苏州市委十一届四次全体（扩大）会议的部署，今年政府工作的总体要求是：认真学习贯彻中共十八大精神，高举中国特色社会主义伟大旗帜，以邓小平理论、“三个代表”重要思想和科学发展观为指导，以转变经济发展方式为主线，以提高经济增长质量和效益为中心，围绕建设“三区三城”总目标，突出创新引领、聚焦转型、生态优先、民生为本等重点，稳中求进，量质并举，努力实现经济社会持续健康发展。

　　全市经济社会发展的主要预期目标是：地区生产总值增长10%，地方公共财政预算收入增长10%，服务业增加值占地区生产总值的比重提高1.5个百分点，新兴产业和高新技术产业产值占规模以上工业产值的比重各提高2个百分点左右，全社会固定资产投资增长15%左右，社会消费品零售总额增长16%左右，进出口总额增长3%左右，实际利用外资与上年基本持平，城镇居民人均可支配收入和农民人均纯收入均增长12%以上，居民消费价格总水平涨幅控制在3.5%左右，城镇登记失业率控制在3.5%以内，全社会研究与试验发展经费支出占地区生产总值的比重达到2.7%，单位地区生产总值能源消耗下降、主要污染物排放量削减完成省下达任务。

　　我们要认真落实十八大确定的“五位一体”的总布局，按照本届政府任期内实现“六个全国领先”的要求，重点把握好以下几个方面：

　　坚定不移地以加快转型升级为核心任务。着力推动增长动力由资源消耗为主向创新驱动为主转变、经济结构由制造业为主向服务经济为主转变、工业经济由传统产业主导向新兴产业主导转变。完善区域创新体系，更多地依靠科技进步、劳动者素质提高和管理创新促进经济发展。改善需求结构，牢牢把握扩大内需这一战略基点，增强消费对经济增长的基础作用，发挥好投资对经济增长的关键作用，千方百计拓展外需、稳定外贸，使消费、投资、出口三者结构更加合理、拉动更加有力。优化产业结构，发展新兴产业，壮大现代服务业，提高农业综合生产能力和综合效益。做大做强中心城市，更好地发挥其对区域发展的辐射带动作用。

　　坚定不移地以推进改革开放为强大动力。坚持改革不停顿、开放不止步，大胆探索、勇于实践，用改革开放的办法解决前进中的矛盾和问题。尊重群众的首创精神，提高改革决策的科学性，增强改革措施的系统性、整体性、协同性。深化经济体制改革，构建充满活力、富有效率、更加开放、有利于科学发展的体制机制。深化行政体制改革，加快政府职能转变，建设职能科学、结构优化、廉洁高效、人民满意的服务型政府。更加注重对外开放与对内开放相结合、引资与引智相结合、“引进来”与“走出去”相结合，加速形成以国际化企业为主体、国际化城市为基础、国际化人才为支撑的新格局。

　　坚定不移地以促进城乡一体为战略追求。深入推进城乡一体化发展综合配套改革，持续缩小城乡差距，实现城乡共同繁荣，把城乡一体化打造成为苏州最大的优势和品牌。推动资源要素向农村配置，优化城乡布局，拓展发展空间，着力形成以工促农、以城带乡、工农互惠、城乡一体的新型工农、城乡关系。贯彻多予少取放活的方针，大力发展农村经济，培育新型经营主体，鼓励农民从就业向创业转变，从单纯生产者向既是生产者又是投资者转变，让广大农民平等参与现代化进程、共同分享现代化成果。

　　坚定不移地以加强社会建设为关键环节。坚持人民主体地位，树立强市惠民理念，更加注重权利公平、机会公平、规则公平，真正让人民群众成为幸福苏州的建设者和共享者。促进城乡居民收入普遍较快增长，深化收入分配制度改革，缩小收入差距，做好就业创业、社会保障、社会救助、优抚安置等工作。健全公共服务体系，竭力解决群众最关心最直接最现实的利益问题，努力让全市人民学有优教、劳有厚得、病有良医、老有颐养、住有宜居。加强和创新社会管理，增强城乡社区服务功能，引导社会组织健康发展，充分发挥群众参与社会管理的基础作用，构建社会主义和谐社会。

　　坚定不移地以打造文化强市为重要使命。加强社会主义核心价值体系建设，激发文化创造力，增强文化软实力、竞争力。弘扬民族精神和时代精神，发扬“张家港精神”、“昆山之路”和“园区经验”，与时俱进丰富发展苏州城市精神。坚持把社会效益放在首位、社会效益和经济效益相统一，推进文化事业全面繁荣、文化产业快速发展。促进文化旅游深度融合，发展新型文化业态，提高文化产业规模化、集约化、专业化水平。以高度的文化自觉和文化自信，推动文化传承、借鉴与创新互动并进。

　　坚定不移地以建设生态文明为长远大计。树立尊重自然、顺应自然、保护自然的理念，把生态文明建设放在更加突出的位置。健全土地开发、资源节约和生态环境保护的体制机制，推进绿色发展、循环发展、低碳发展，实现经济效益和生态效益的协调统一。加强“绿色苏州”建设，实施重大生态修复工程，维护生态安全，增强生态产品生产能力。注重宣传教育，提高全体市民的节约意识、环保意识、生态意识。始终不渝地走生产发展、生活富裕、生态良好的可持续发展之路，建设美丽宜居新苏州。

　　三、2013年主要任务

　　新的一年里，我们力求在以下十个方面取得新成效。

　　1．在推动经济持续增长上取得新成效

　　紧紧抓住国际环境倒逼我们扩大内需、提高创新能力、加快转变经济发展方式的新机遇，保持消费、投资、出口协调增长，力促经济平稳较快发展。强化有效投入的拉动作用，集中力量建设总投资超过1万亿元的200项重大项目，年内完成投资1900亿元以上。着力化解土地、资金等方面的突出矛盾，保障项目顺利推进。充实项目储备，增强投资后劲。优化商贸网点布局，打造中心商圈，做强特色商业街区，构建轨道交通站点商贸集群，深入实施社区商业“双进工程”和“万村千乡市场工程”，创建电子商务示范基地和示范企业，发展新型商贸业态。全面提升旅游业，扩大旅游消费，建设文化旅游示范区、旅游度假区和重点旅游项目，开展智慧旅游城市试点工作，力争吴中太湖旅游区和常熟沙家浜虞山尚湖旅游区成为国家5A级景区。积极创建全国文化产业示范区，建设姑苏区文化创意产业集聚区、吴江区丝绸文化旅游产业集聚区和相城区国家数字出版基地，努力将中国苏州文化创意设计产业交易博览会打造成为全国知名的品牌展会，推动文化产业提速发展，促进文化消费。发挥外贸在稳增长中的重要作用，分类指导企业拓展国际市场，支持出口型生产企业和大型商贸流通企业建立国际营销网络。转变外贸发展方式，建设国家外贸转型升级专业型示范基地，提高一般贸易比重，引导加工贸易向价值链两端提升、向总部经济形态发展，扩容提档服务贸易，推动软件和服务外包产业实现新突破。鼓励拥有自主知识产权和自主品牌的商品出口，促进商品个性化、出口多样化。有效应对国际贸易摩擦，推广出口信用保险，防范和化解贸易风险。

　　2．在增强产业竞争能力上取得新成效

　　坚持创新引领，更加注重结构、质量和效益，构建现代产业新体系。大力发展新兴产业和高新技术产业，延伸完善纳米新材料、新型平板显示、新能源、高端装备制造、生物医药、软件和集成电路等重点产业链，建设一批位居全省乃至全国行业首位的特色产业基地、产业集群。继续实施万企升级计划，推动企业技术改造，转移产业低端环节，促进支柱产业和传统产业优化升级。把稳增长、增实力的关键放到稳住实体经济上，支持企业调结构、转方式、抓创新、强管理。培育一批规模较大、创新能力较强的骨干企业，增强大企业的支撑带动作用。以信息技术集成应用为重点，深入创建工业化与信息化融合示范区、示范企业。促进服务业比重提高、质量提升，重点发展金融、商务、科技和知识产权服务等生产性服务业，打造苏州工业园区国际商务区、苏州高新区国家知识产权服务业发展试验区和昆山东部新城金融街，高水平规划建设现代物流园、软件产业园、工业设计产业园，扶持培育在技术、业态、品牌等方面具有领先优势的创新型服务业企业。积极开展服务业综合改革试点，新增1～2个省级现代服务业示范区。强化农业生态、生产、生活、生物功能定位，落实优质水稻、特色水产、高效园艺、生态林地“四个百万亩”空间布局，加强农业综合生产能力建设，大力发展规模农业、设施农业、生态农业，新增现代农业园区面积6.7千公顷。增强自主创新能力，争创国家创新型城市。强化企业创新主体地位，加强科技创新、产品创新、品牌创新、产业组织创新、商业模式创新，推动大中型内资工业企业全部建立研发机构，新增一批国家级企业技术中心和技术创新示范企业。拓展科技资源共享、科技成果转化和科技金融服务平台，建设国家科技成果转化服务示范基地。深化产学研合作，鼓励骨干企业与科研机构、高等院校组建产业技术创新战略联盟。扩大“科贷通”覆盖范围，做好国家促进科技金融结合试点工作。加强知识产权保护，培育拥有优势品牌、驰名商标、重要版权、发明专利的知识产权密集型企业。加强新兴产业重要技术标准研制，新增国际、国家标准化组织2个以上。深入实施“1010”工程、姑苏人才计划、海鸥计划，办好“千人计划”太湖峰会、苏州国际精英创业周、技能英才周、“赢在苏州”创新创业大赛，大力引进能够引领和支撑产业发展的领军人才与创新团队。

　　3．在深化改革扩大开放上取得新成效

　　推进重要领域改革，提升经济国际化水平。优化国有经济资本结构、产业结构，增强国有企业发展活力。降低民间投资准入门槛，扶持发展成长性中小民营企业，积极培育规模型民营企业。落实过渡性财政扶持措施，确保营业税改征增值税工作顺利进行。推动金融创新，支持企业上市，争取“新三板”扩容试点和跨国公司外汇资金集中运营试点。引进各类金融机构，发展壮大地方法人金融机构。深化事业单位分类改革。实施县级公立医院综合改革，有序推进医药分开。稳步开展行业协会改革试点，支持公益性社会组织发展。以经济国际化引领开放提升，在转型创新中汇聚优质资源。提高利用外资水平，重点引进新兴产业、现代服务业龙头项目和高端业态项目。创新体制机制，优化提升功能，加快开发区转型发展步伐。放大中新合作优势，争创开放创新示范区，更大力度推进苏州工业园区—相城区合作经济开发区建设和招商。推动建立昆山深化两岸产业合作试验区、太仓港综合保税区。扩大进口贸易，完善张家港保税港区汽车整车进口口岸功能，力争将中国国际进口产品交易会办成国际知名展会。把“走出去”作为对外开放的重大举措，引导企业通过境外加工贸易、境外并购参股、资源开发合作、工程总承包等方式参与海外市场竞争，培育本土跨国公司。发挥外事、侨务、对台工作的重要作用，推进地方公共外交和人文交流。加快南北共建园区建设步伐，做好援藏、援疆等对口援建工作。

　　4．在推进城乡一体发展上取得新成效

　　加大城乡统筹力度，在形态优化和政策创新上实现更大突破。完善城乡一体发展规划，促进城镇建设、土地利用、产业发展、生态建设等规划有机融合。更大范围落实城乡建设用地置换空间，用足用好城乡建设用地增减挂钩政策，开展土地复垦整治，合理配置产业用地。抓住国家重点推进城镇化的有利时机，继续推动农民居住向新型社区集中、工业企业向工业园集中、耕地向规模经营集中，鼓励农户将集体资产所有权、土地承包经营权、宅基地及住房置换成股份合作社股权和城镇住房，引导更多的农民换股、换保、换房进城进镇落户，加快农民市民化步伐。创新农业生产经营体制，推动农村新型合作经济组织规范发展、异地发展、联合发展，支持农村集体经济参与工业化、信息化、城镇化和农业现代化建设，农村集体资产和村均收入均增长12%以上。强化集体经济资源、资产、资金监管，稳步推进集体产权制度改革。进一步实施各项强农惠农富农政策，促进城乡要素平等交换、公共资源向农村倾斜，加快实现城乡基本公共服务均等化。广辟农民增收渠道，健全长效增收机制。增强农业担保能力，探索扩大政策性农业保险领域。改造农村水利设施，提高防洪抗旱能力。加强古村落保护和管理，激发古村落发展活力。巩固村庄环境整治成果，落实长效化、专业化、社会化管理措施，持续改善农村环境状况。

　　5．在加强城市建设管理上取得新成效

　　完善城市功能，增强承载能力，美化城市形象。按照“一核四城”定位，启动新一轮城市总体规划和综合交通规划编制工作，抓紧制定重点片区、重大工程规划，做强做精做美古城核心，加快建设东部综合商务城、西部生态科技城、南部太湖新城和北部高铁新城。把姑苏区打造成为历史文化保护示范区、高端服务经济集聚区、文旅融合发展创新区、和谐社会建设样板区；把吴江区建设成为南部现代新城区、优势产业新板块、开放创新新高地、和谐乐居新家园。推动沪通、沿江、通苏嘉城际铁路建设，完成苏州火车站综合改造任务。建成太仓港疏港高速公路，续建常嘉高速公路昆山至吴江段、张家港疏港高速公路，实施312国道苏州段分流线工程。建设中环快速路及东环、南环、西环快速路延伸线，扩建相城大道。确保轨道交通2号线投入试运营，推进4号线及支线、2号线延伸线和国内首条现代有轨电车线路建设。加快建设太仓港集装箱码头四期工程。加大苏南运河苏州段、杨林塘、申张线三级航道整治力度。建立人口基础数据库，扩大无线局域网热点覆盖面，推动家庭信息化。新增人防设施75万平方米。确保餐厨垃圾收集处置二期工程投入使用，实施建筑垃圾资源化利用终端处置项目。大力开展整治乱停乱放、非法营运、小区环境、违法建设“四大行动”，落实“路长制”和“门前三包”责任制，全面提升交通秩序、运管服务、人居环境、市容市貌。推行数字化城市管理，提高管理效能。完善市政设施，加强日常保洁和管护，塑造整洁优美的城市形象。

　　6．在强化生态环境建设上取得新成效

　　认真贯彻节约优先、保护优先、自然恢复为主的方针，更加自觉地珍惜自然、爱护生态。加强集中式饮用水源地水质监测和蓝藻、水草防控，基本完成全市同城第二水源地建设和自来水厂深度处理工艺改造任务，确保饮用水安全。推进太湖流域水污染治理和东太湖北部湖区生态清淤，建设七浦塘和永昌泾综合整治工程。实施古城区河道“活水工程”，疏浚整治农村河道。建设污水处理厂，完善污水收集系统，实现污泥无害化处置。建立健全水资源开发利用、用水效率控制、水功能区限制纳污“红线”制度，提高工业污水达标排放水平和水环境功能区水质达标率。治理机动车尾气、工业废气和扬尘污染，改善空气质量。控制噪声污染，严肃查处噪声扰民行为。严格防治重金属污染，规范处置利用工业固体废物。推行生活垃圾分类收集，实现城区生活垃圾全量焚烧和餐厨垃圾资源化利用。实行环境风险源分类分级动态管理，有效预防和处置环境风险事故。把化解产能过剩和加强节能减排结合起来，深入开展“万家企业节能低碳行动”和“能效之星”创建活动，促进节能降耗，削减排污总量。实施循环经济示范工程，争创国家循环经济示范市。严格保护基本农田，积极盘活存量土地，提高土地节约集约利用水平。制定生态文明建设规划，抓好全国生态文明城市试点和国家低碳城市试点工作。完善生态补偿制度，深化资源性产品价格和环保收费改革。大力推进环古城河风貌保护提升和大运河、胥江综合整治工程。建设上方山森林植物园、动物园、游乐园及石湖景区二期工程，持续开展太湖、阳澄湖生态修复。加快实施虎丘湿地公园等重大绿化工程，市区新增绿地500万平方米。加强农田河湖林网、绿色通道和生态片林建设，农村新增林地绿地4.2千公顷，全市陆地森林覆盖率达到28.6%。

　　7．在统筹发展社会事业上取得新成效

　　推动各项事业全面进步，努力提供优质公共服务。落实中长期教育改革和发展规划纲要，办好人民满意的教育。促进城乡教育一体化发展，基本建成省义务教育优质均衡改革发展示范区，力争成为全国首个义务教育发展基本均衡地级市。新建和改扩建幼儿园100所。深化素质教育，加强教育督导，实施学生身心健康关爱工程，探索建立多元化评价体系，让学生得到全面发展。高度重视校长和教师队伍建设，构筑教育人才高地。推进教育综合改革，开展现代学校制度试点，鼓励发展民办教育，进一步规范社会机构办学行为。实施职业教育能力创新提升工程，构建现代职业教育体系。支持高等教育改革发展，加强与在苏高校的合作，促进科教协同创新。倡导终身学习，提高全民素质。面向群众文化需求，增强文化服务能力，确保国家公共文化服务体系示范区创建达标。加大历史文化名城名镇保护力度，推进虎丘地区综合改造、桃花坞历史文化片区综合整治保护利用和天赐庄片区、渔家村越城遗址、同里历史文化遗产保护整治等工程，保护和修复古典园林，修缮利用古建老宅。建设中国昆曲剧院、中国非物质文化遗产展示中心、苏州图书馆二期和文化资源数字化工程，打造博物馆城。抓好大运河申报世界文化遗产各点段环境整治工作，积极申报创意城市—工艺美术之都。繁荣文艺创作，办好重大文化活动。基本完成新一轮《苏州市志》编纂任务。加快建设市档案馆新馆。优化公共卫生资源配置，镇（街道）全部建立政府办社区卫生服务中心。改造升级社区卫生机构信息系统，建立社区临床检验集中检测中心。规范管理民营医疗机构。建成苏州大学附属第一医院门诊急诊楼、市立医院本部门诊急诊楼，抓紧建设苏州大学附属儿童医院园区总院，开工建设市公共医疗中心，筹建吴门医派研究院。增设便民体育健身设施，推动全民健身活动广泛开展。建成市全民健身中心体育场和市网球中心、儿童业余体校，筹建运河体育公园、体育博物馆。健全人口与家庭发展公共服务和政策体系，做好计划生育家庭特别扶助对象帮扶工作，实现免费孕前优生健康检查全覆盖。贯彻男女平等基本国策，保障妇女儿童合法权益，保持我市妇女儿童事业率先发展。坚持走军民融合式发展路子，提高国防动员和后备力量建设质量，开展争创全国双拥模范城“六连冠”工作。

　　8．在不断改善人民生活上取得新成效

　　增加民生投入，办好民生实事，提高民生质量。更大力度落实城乡居民收入六年倍增计划，努力实现居民收入增长和经济发展同步、劳动报酬增长和劳动生产率提高同步。贯彻就业优先战略和更加积极的就业政策，重点做好就业困难人群的就业工作，确保城乡零就业和零转移贫困家庭动态清零。深化国家创业型城市建设，以创业促进就业，推动实现更高质量的就业。持续开展城乡劳动力职业培训，完善城乡一体的人力资源市场。加强劳动保障监察和争议调解仲裁工作，推动企业建立工资集体协商制度，巩固和谐劳动关系。推进城乡社会保障一体化，稳步提高待遇标准。探索引入商业保险参与社会保障体系建设。构建适度普惠、城乡一体、公平公正的社会救助体系，保持救助标准全国领先。发展红十字和公益慈善事业。合理布局养老服务设施，推广“虚拟养老院”模式，扩大居家养老服务对象范围。建设市残疾人康复训练服务中心二期工程，提升康复服务水平。加大保障性住房建设力度，促进廉租住房与公共租赁住房统筹建设、并轨运行。完成南环新村危旧房解危改造工程和城区居民家庭“改厕”任务，推进第二批危旧房改造。继续开展老住宅小区和零星居民楼综合整治，解危修缮直管公房，改造城中村（无地队）。拓宽住房公积金制度保障面，力争城镇职工住房公积金覆盖率达到80%。加快公交优先进程，拓展网络，增加车辆，优化服务，提高乘坐便捷性、舒适性。做好苏州社会保障·市民卡启用和功能拓展工作。深入开展放心消费创建活动，加强食品安全监测、检验和防控，争创国家和省药品安全示范市（区）。扩建“菜篮子”生产基地，改造农贸市场，新增一批农产品配送中心、直供网点和放心粮油店。健全粮食市场体系，完成地方粮食储备任务。加强价格调控和监管，保持物价水平总体稳定。

　　9．在促进社会和谐稳定上取得新成效

　　加强精神文明、民主法治和平安苏州建设，维护安定和谐大局。推进精神文明创建活动和社会公共文明建设行动，定期进行城市文明程度指数测评，健全城乡联动、文明共建、长效管理机制，争创全国文明城市群。实施基层科普行动计划，提高市民科学素养。支持哲学社会科学研究，推动形成一批高质量的成果。坚持依法治市，争创全省全国法治建设先导区，建成一批民主法治示范村、示范社区。积极开展“六五”普法，扩大法律援助受惠面。建设企业和个人信用平台，构建社会诚信体系。实施社会管理创新重点项目，着力形成源头治理、动态管理、应急处置相结合的管理机制。抓好社会管理综合治理，依法打击违法犯罪活动，有效预防和惩处电信诈骗、非法集资、侵犯知识产权和网络违法犯罪等行为，高度警惕和坚决防范敌对势力的渗透破坏活动。做好刑释解教人员安置帮教和社区矫正工作。改进流动人口和特殊人群服务管理。健全重大决策社会稳定风险评估机制和矛盾纠纷排查化解长效机制，完善基层信访网络，认真处理群众来信来访。强化基层应急管理，增强应急救援能力。强化消防和森林防火工作。加强公共安全体系和企业安全生产基础建设，严格落实安全生产责任制和各类防范措施，坚决防止重特大安全事故发生。

　　10．在提高政府工作水平上取得新成效

　　牢记全心全意为人民服务的宗旨，坚持以人为本、执政为民，切实加强自身建设，不断改进政府工作。实施第六轮行政审批制度改革，稳妥推进省直管县体制改革试点和经济发达镇行政管理体制改革试点，健全“区镇合一”管理体制。加强法治政府建设，切实做到依法履行职责、行使权力。高质量起草地方性法规、制定政府规章，提高立法后评估成效。完善规范性文件备案审查制度，实行定期清理和通报。全面落实行政执法责任制，做到公正、严格、文明执法。做好行政复议和应诉工作。优化行政服务中心、便民服务中心功能，建立公共资源交易中心。认真开展第三次经济普查。严格遵守廉洁从政的各项规定，完善惩治和预防腐败体系，严肃查处违法违纪案件，努力实现干部清正、政府清廉、政治清明的目标要求。健全权力运行制约和监督体系，保证人民赋予的权力始终用来为人民谋利益。认真执行中共中央和省委、市委关于改进工作作风、密切联系群众的各项规定，深入推进机关作风效能建设，坚决整治庸懒散奢等不良风气。加强调查研究，扎实开展以访基层企业促转型升级、访基层组织促创先争优、访基层群众促改善民生为主要内容的下基层活动，着力解决基层和群众反映强烈的热点、难点问题。规范各类论坛、庆典、研讨会等活动，精简会议和文件简报，改进会风文风。深化政务公开，持之以恒开展纳税人评议政风行风活动，切实纠正损害人民利益的不正之风。严格财政预算管理，控制因公出国（境）、公务接待、公务用车购置等经费支出，稳妥推进公务用车改革。强化预算执行审计、经济责任审计、政府投资跟踪审计和专项资金绩效审计，对会议活动等经费使用情况进行审查，充分发挥审计监督作用。各级政府要严格执行人民代表大会及其常务委员会的决定和决议，主动接受人大的法律监督、工作监督和政协的民主监督，接受司法机关的监督，接受新闻媒体和社会舆论的监督。认真实施人大议案，提高代表建议和政协提案的办理质量。

　　各位代表，展望新的一年，困难和挑战考验着我们，责任和使命激励着我们。让我们紧密团结在以习近平同志为总书记的党中央周围，高举中国特色社会主义伟大旗帜，全面贯彻落实十八大精神，在中共苏州市委的坚强领导下，同心同德，锐意进取，扎实工作，奋力开创改革开放和现代化建设的新局面！
